# The Synthesis, Structure, and Properties of a Polynitro Energetic Complex with a Hexaamminecobalt(III) Ion as a Stabilizing Core

**DOI:** 10.3390/ma18133004

**Published:** 2025-06-25

**Authors:** Zhiwei He, Feng Yang, Xianfeng Wang, Ming Lu

**Affiliations:** 1 School of Chemical and Blasting Engineering, Anhui University of Science and Technology, Huainan 232001, China; 2 School of Chemistry and Chemical Engineering, Nanjing University of Science and Technology, Nanjing 210094, China; wangxf2519@163.com (X.W.); luming302@126.com (M.L.)

**Keywords:** energetic complexes, crystal structure, thermal stability

## Abstract

Energetic complexes with multi-component architectures represent a frontier in contemporary energetic materials research. In this work, we report a novel high-energy complex—bis(5-nitro-3-(dinitromethyl)-1,2,4-triazole)-hexaamminecobalt(III) [[Co(NH_3_)_6_](HNTD)(NTD)·H_2_O]—that is synthesized using the oxygen-rich energetic compound 5-nitro-3-(trinitromethyl)-1,2,4-triazole (HNTF) as a precursor. Compared with metallic H_2_NTD salts, [Co(NH_3_)_6_](HNTD)(NTD)·H_2_O exhibits a higher density (*ρ* = 1.886 g cm^−3^) and unrivaled energy properties (*V*_d_ = 8030 m s^−1^ and *P* = 29.2 GPa). The formation of a dense hydrogen-bonding network—mediated by ammonium groups in the [Co(NH_3_)_6_]^3+^ core and nitro groups of HNTD^−^ and NTD^2−^—significantly dampens the mechanical sensitivity (*IS* = 10 J and *FS* = 140 N). These combined attributes establish [Co(NH_3_)_6_](HNTD)(NTD)·H_2_O as a promising high-energy-density material (HEDM), offering critical insights for the design of next-generation energetic complexes.

## 1. Introduction

Energetic materials, which are distinguished by their extraordinarily high energy-release rates and the simultaneous generation of significant acoustic, optical, and thermal effects during energy discharge, have seen extensive adoption across civilian and military domains since their inception [1,2,3,4,5].

**Figure 1:**
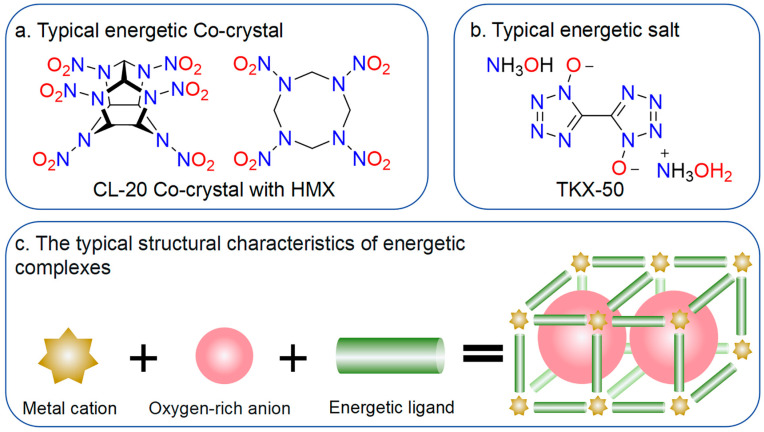
Some typical representatives of the methods for preparing energetic materials with a low sensitivity.

In recent years, the advancement of target application fields and innovations in material synthesis methodologies have driven the compositional diversification of energetic materials, accompanied by continuous improvements in their explosive performance [6,7,8,9]. Undoubtedly, the development of high-energy materials with inherent safety remains a central focus in energetic materials research, irrespective of their application scenarios (e.g., primary explosives or high-energy explosives). Being widely acknowledged, ionic energetic compounds and co-crystalline materials have emerged as promising strategies for designing high-stability, high-energy compounds (Figure 1) [10,11,12,13,14,15,16,17]. However, these approaches are challenged by fundamental limitations, such as density degradation and the scarcity of structurally robust co-crystals, which collectively hinder the rapid development of novel high-energy–high-stability energetic compounds. Against this scientific backdrop, a novel multicomponent architectural design—integrating the advantages of dense hydrogen-bonding networks in ionic compounds and the high-density features of MOF-type materials—has been explored for synthesizing high-stability–high-energy systems, specifically energetic coordination complexes [18,19,20,21,22,23]. These complexes exhibit remarkable diversity in physicochemical properties, which is attributed to the synergistic effects of abundant intracomplex hydrogen-bonding networks, coordination bonding, and the tunable nature of ligand/metal ion combinations. With the increasing prevalence of in-depth research into energetic complexes, their applications have expanded to primary explosives, ammonium perchlorate decomposition catalysts, and high-energy low-sensitivity explosives. Notably, previous studies predominantly employed oxygen-rich oxyacid anions or nitrogen-rich anions as primary ligands—a strategy that often compromises the oxygen balance of the complexes and consequently limits their maximum detonation performance.

Building upon the aforementioned research landscape, we synthesized a novel high-energy complex, [Co(NH_3_)_6_](HNTD)(NTD)·H_2_O, utilizing 5-nitro-3-(trinitromethyl)-1,2,4-triazole (HNTF) as the starting material. The complex demonstrates a low mechanical sensitivity, which is attributed to the dense hydrogen-bonding network within its crystal lattice. Notably, although the structure of the complex contains a large number of ammonia molecules, the inherent high density of both 5-nitro-3-(dinitromethyl)-1,2,4-triazole (H_2_NTD) and cobalt ions collectively imparts the complex with a relatively high overall density. This study not only enriches the research scope of high-energy complexes but also provides a reference for the development of novel low-sensitivity energetic materials.

## 2. Results and Discussion

### 2.1. Synthesis

**Scheme 1:**
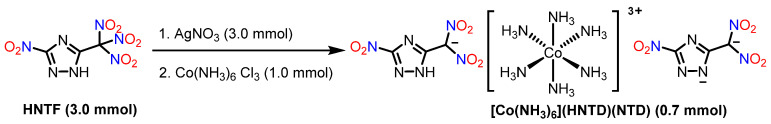
The synthetic route of compound [Co(NH_3_)_6_](HNTD)(NTD).

The synthetic pathway for complex [Co(NH_3_)_6_](HNTD)(NTD)·H_2_O is outlined in Scheme 1. Equimolar amounts of compound HNTF [24] and silver nitrate were stirred in deionized water at room temperature for 12 h. The resulting precipitate was collected by filtration to afford the intermediate (NTF-Ag). This intermediate was then reacted with Co(NH_3_)_6_Cl_3_ in deionized water under stirring for 8 h. The reaction mixture was filtered and the filtrate was evaporated to yield complex [Co(NH_3_)_6_](HNTD)(NTD)·H_2_O.

### 2.2. Crystal Structures

Similarly to most energetic compounds bearing highly labile N-H protons, HNTF exhibits a pronounced hygroscopicity, rendering it difficult to isolate corresponding crystals via the evaporation of its alcoholic, ethyl acetate, or acetonitrile solutions. To obtain detailed crystallographic data for HNTF, we employed water-immiscible dichloromethane as the solvent and performed a slow evaporation of its dichloromethane solution within a desiccator, ultimately achieving the growth of suitable single crystals.

**Figure 2:**
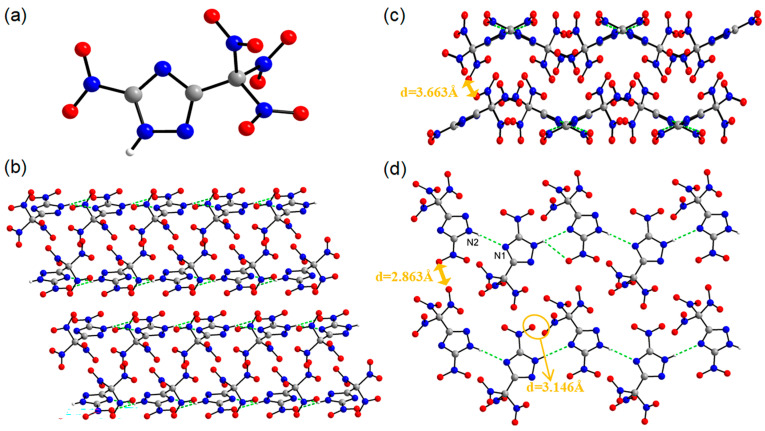
(**a**) The molecular structure of HNTF. (**b**) The crystal packing diagram of HNTF. (**c**) The arrangement of intermolecular layers. (**d**) The arrangement of molecules within the layer and the distribution of hydrogen bonds.

Although HNTF does not exhibit a planar geometry (Figure 2a), its molecular packing forms highly ordered wave-like layered stacks (Figure 2b,c). The nitro groups, which are positioned at both termini of the molecular framework, facilitate the close proximity of nitro moieties during stacking. Notably, the minimum distance between nitro oxygen atoms in interlayer molecules is 3.662 Å (Figure 2c), whereas the intralayer distance is significantly shorter at 2.863 Å (Figure 2d). Such a compact nitro group arrangement is typically correlated with an increased mechanical sensitivity in energetic materials. Additionally, due to the single hydrogen atom in the molecule, HNTF crystals contain a limited number of hydrogen bonds. However, it is noteworthy that the crystal density of HNTF at room temperature is 1.95 g/cm^3^, which aligns well with the reported experimental value (1.94 g/cm^3^ [24]).

**Figure 3:**
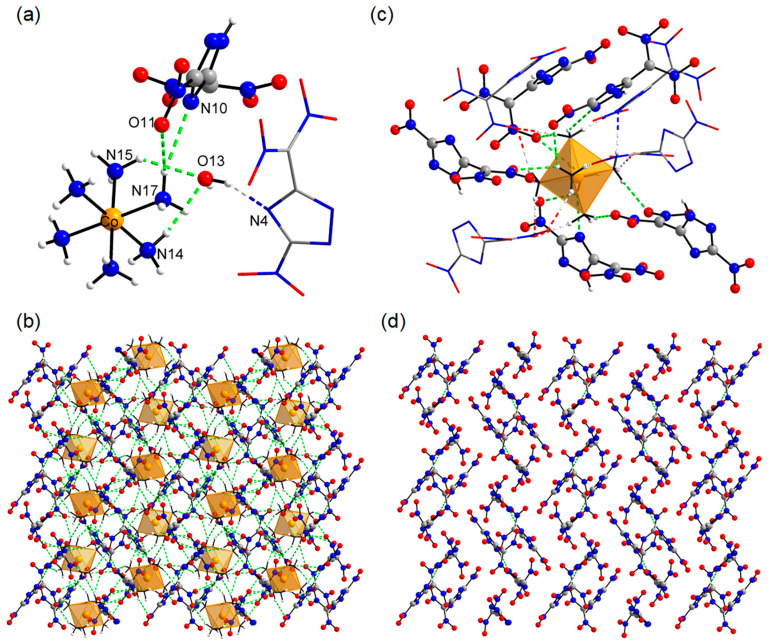
(**a**) The structure of [Co(NH_3_)_6_](HNTD)(NTD)·H_2_O (HNTD^−^ and NTD^2−^ are represented by ball-and-stick and stick models, respectively). (**b**) The crystal packing diagram of [Co(NH_3_)_6_](HNTD)(NTD)·H_2_O. The yellow polyhedron is Co(NH_3_)_6_; HNTD^−^ and NTD^2−^ are represented by ball-and-stick and stick models, respectively. (**c**) The hydrogen bond connection situation between [Co(NH_3_)_6_]^3+^ and the surrounding HNTD^−^ and NTD^2−^. The yellow polyhedron is Co(NH_3_)_6_. (**d**) The packing diagram excluding [Co(NH_3_)_6_]^3+^, i.e., the packing diagram of only the HNTD^−^ and NTD^2−^ part. The green dotted lines represent hydrogen bonds.

Crystallographic analysis reveals that in [Co(NH_3_)_6_](HNTD)(NTD)·H_2_O, coordination bonds and hydrogen bonds alternately link cobalt ions, ammonia molecules, and energetic (HNTD^−^ and NTD^2−^) moieties, giving rise to a distinctive “core–shell structure” within the unit cell (Figure 3c). In this architecture, nine high-energy ions (among them, there are five HNTD^−^ and four NTD^2−^) encapsulate a non-energetic Co(NH_3_)_6_ core. Notably, energetic moieties exist in two valence states within the structure—monovalent C_3_N_6_O_6_H (HNTD^−^) and divalent C_3_N_6_O_6_ (NTD^2−^) (Figure 3a). This valence diversity facilitates the formation of a limited hydrogen-bonding network among HNTD^−^ and NTD^2−^ (Figure 3d). Despite HNTD^−^ and NTD^2−^ possessing one fewer nitro group than HNTF, as well as the inclusion of one crystallization water molecule in the [Co(NH_3_)_6_](HNTD)(NTD)·H_2_O crystal, its room-temperature density reaches 1.886 g/cm^3^.

### 2.3. Thermal Stability and Mechanical Sensitivity

**Figure 4:**
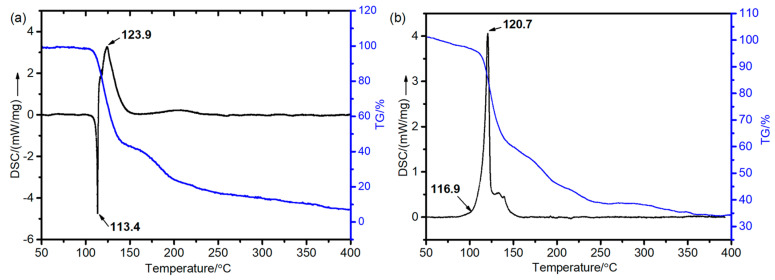
(**a**) The TG-DSC curve of the compound HNTF. (**b**) The TG-DSC curve of the compound [Co(NH_3_)_6_](HNTD)(NTD)·H_2_O.

Thermal stability is a critical parameter for evaluating the stability of energetic compounds. In this study, the thermal decomposition behaviors of HNTF and [Co(NH_3_)_6_](HNTD)(NTD)·H_2_O were characterized using TG-DSC under a nitrogen atmosphere at a heating rate of 5 °C min^−1^ (Figure 4). The DSC curve of HNTF reveals two distinct thermal stages—an endothermic phase followed by an exothermic phase. The corresponding TG analysis indicates that HNTF initiates decomposition at the endothermic peak, with an initial decomposition temperature of 113.4 °C. In contrast, [Co(NH_3_)_6_](HNTD)(NTD)·H_2_O exhibits a single exothermic decomposition process, with an initial decomposition temperature of 116.9 °C, which is 3.5 °C higher than that of HNTF. Notably, the presence of the heavy metal Co in [Co(NH_3_)_6_](HNTD)(NTD)·H_2_O results in a significantly higher solid residue (34.5%) after complete decomposition compared to HNTF.

**Figure 5:**
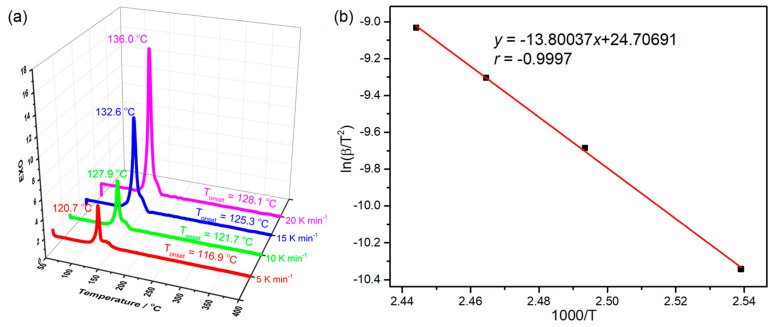
(**a**) DSC curves of [Co(NH_3_)_6_](HNTD)(NTD)·H_2_O at different heating rates. (**b**) Thermodynamic equations fitted using the Kissinger method.

Furthermore, non-isothermal kinetic studies were carried out at four different heating rates to obtain the sensitivity data, including the thermal explosion temperature (*T*_pb_), the self-accelerating decomposition temperature (*T*_SADT_), and the activation energy (*E*), of [Co(NH_3_)_6_](HNTD)(NTD)·H_2_O to temperature changes (Figure 5). The experimental results show that the activation energy (calculated using Kissinger’s method) of the compound [Co(NH_3_)_6_](HNTD)(NTD)·H_2_O is *E_k_* = 114.7 kJ/mol (*SE* = 0.16), and the *T*_SADT_ and *T*_pb_ are 110.5 and 110.7 °C, respectively. These data indicate the adaptability of the compound [Co(NH_3_)_6_](HNTD)(NTD)·H_2_O under the application environmental conditions of energetic compounds.

In terms of mechanical sensitivity, the impact sensitivity (IS) and friction sensitivity (FS) of HNTF measured using the standard BAM impact sensitivity tester and friction sensitivity tester are 4 J and 80 N, respectively. The impact sensitivity and friction sensitivity of [Co(NH_3_)_6_](HNTD)(NTD)·H_2_O are 10 J and 140 N, respectively, which shows that its mechanical sensitivity is significantly lower compared to HNTF.

### 2.4. Theoretical Analysis of Stability

**Figure 6:**
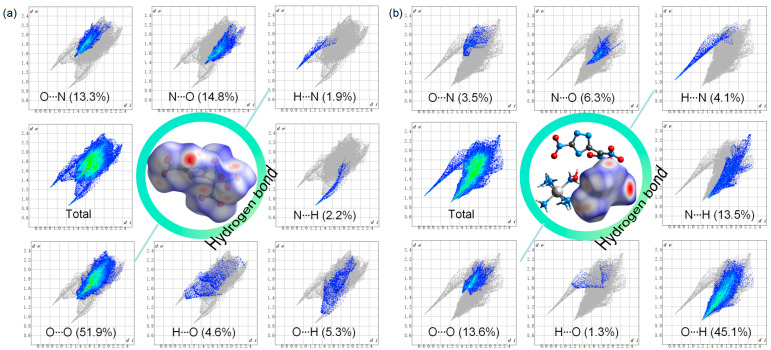
Heathfield and fingerprint diagrams of compounds HNTF (**a**) and [Co(NH_3_)_6_](HNTD)(NTD)·H_2_O (**b**).

To theoretically rationalize the substantial stability disparity between HNTF and [Co(NH_3_)_6_](HNTD)(NTD)·H_2_O, Hirshfeld surface [25] analysis coupled with fingerprint plots was employed to characterize atomic-level interactions. For HNTF, hydrogen-bonding interactions (H···O/N) constitute a minor fraction of the total intermolecular forces, whereas O···O interactions dominate at 51.9%—a direct consequence of densely packed nitro groups (Figure 6a). This nitro–nitro interaction dominance mechanistically explains HNTF’s low stability, as close-proximity oxygen atoms in nitro groups enhance electrostatic repulsion and thermal sensitivity [26,27].

In contrast, [Co(NH_3_)_6_](HNTD)(NTD)·H_2_O exhibits a 50% increase in H···O/N interactions, which is indicative of a more extensive hydrogen-bonding network. The [Co(NH_3_)_6_]^3+^ core introduces steric hindrance, physically separating HNTD^−^ and NTD^2−^ anions and reducing O···O interactions to 13.6% (vs. 51.9% in HNTF) (Figure 6b). This structural confinement diminishes nitro group clustering, thereby dampening mechanical and thermal sensitivities. The synergistic effects of enhanced hydrogen bonding and steric shielding from the cobalt complex collectively stabilize the crystal lattice, rationalizing the superior stability of [Co(NH_3_)_6_](HNTD)(NTD)·H_2_O relative to HNTF.

### 2.5. Detonation Performance

For energetic materials, density, molecular composition, enthalpy of formation (Δ*_f_H*), and oxygen balance (OB) are critical metrics for evaluating detonation performance. In this study, the Δ*_f_H* values of HNTF and [Co(NH_3_)_6_](HNTD)(NTD)·H_2_O were determined using the isodesmic equation and combustion calorimetry, respectively, (detailed experimental protocols are provided in the Supporting Information). The detonation velocity and pressure (Table 1) of the two compounds were calculated using EXPLO5 v6.05.04 software [28] and the K-J equation, respectively, incorporating the experimentally measured molecular composition, density, and enthalpy of formation data.

The oxygen balance of [Co(NH_3_)_6_](HNTD)(NTD)·H_2_O is relatively low at −28.7%, primarily due to two key factors. Firstly, the energetic ion (HNTD^−^ and NTD^2−^) in [Co(NH_3_)_6_](HNTD)(NTD)·H_2_O lacks one nitro group compared to HNTF; Secondly, the [Co(NH_3_)_6_]^3+^ moiety consumes a significant amount of oxygen atoms. Nevertheless, this value remains higher than that of most energetic complexes. Additionally, attributed to the inherently high energy performance of the HNTD^−^ and NTD^2−^ moieties and the high density of [Co(NH_3_)_6_](HNTD)(NTD)·H_2_O, the compound exhibits an excellent detonation performance. Its detonation velocity and detonation pressure reach 8030 m s^−1^ and 29.2 GPa, respectively, which are significantly higher than those of most energetic complexes.

## 3. Conclusions

In general, in this study, a novel polynitro energetic complex—[Co(NH_3_)_6_](HNTD)(NTD)·H_2_O—was prepared using the oxygen-rich and high-energy compound HNTF as the raw material. Its structure, stability, and energy performance were investigated. As a polynitro energetic complex, [Co(NH_3_)_6_](HNTD)(NTD)·H_2_O exhibits a good stability (*IS* = 10 J and *FS* = 140 N) and a higher energy performance (*V*_d_ = 8030 m s^−1^ and *P* = 29.2 GPa) compared with complexes that use non-energetic acid radicals as the oxygen-rich structure. Theoretical studies have shown that in [Co(NH_3_)_6_](HNTD)(NTD)·H_2_O, on the one hand, the [Co(NH_3_)_6_]^3+^ structure hinders the formation of intermolecular nitro interactions that are likely to lead to high mechanical sensitivity. On the other hand, with NH_3_ serving as the intermediate layer of the core–shell structure, the energetic ions HNTD^−^ and NTD^2−^ are connected together to form an energetic shell through a tight hydrogen-bonding network, thereby enabling [Co(NH_3_)_6_](HNTD)(NTD)·H_2_O to have a relatively low mechanical sensitivity while ensuring a high energy performance.

## Figures and Tables

**Table 1:** Physicochemical parameters and detonation performance parameters of compound HNTF, [Co(NH_3_)_6_](HNTD)(NTD)·H_2_O, and some energetic complexes.

Comp.	*ρ* [a]	*T*_d_ [b]	*OB* [c]	*V*_d_ [d]	*P* [e]	*IS* [f]	*FS* [g]
**HNTF**	1.94	113.4	9.1	8983	35.5	4	80
**[Co(NH_3_)_6_](HNTD)(NTD)·H_2_O**	1.88	116.9	−28.7	8030	29.2	10	140
[Ag_7_(tza)_3_(Htza)_2_(H_2_tza)]_*n*_ [29]	2.56	250.0	–33.8	8089	35.0	40	353
[Ag(atrz)_1.5_(NO_3_)]_*n*_ [30]	2.16	257.0	–50.0	7773	29.7	30	-
[Ag(ATCA)ClO_4_]_*n*_ [31]	2.53	230.0	–18.3	6.80	-	5	72
[Cu(ClO_3_)_2_(2,2-dtp)_2_] [32]	1.82	176.0	−59.5	-	-	1	5

a. The measured density. b. Onset decomposition temperature (5K min^−1^). c. Oxygen balance (calculated based on CO_2_). d. Detonation velocity. e. Detonation pressure. f. Friction sensitivity. g. Impact sensitivity.

## Data Availability

The original contributions presented in this study are included in the article/Supplementary Materials. Further inquiries can be directed to the corresponding authors.

## Supplementary Materials

The following supporting information can be downloaded at https://www.mdpi.com/article/10.3390/ma18133004/s1. References [33,34,35,36] are cited in the Supplementary Materials.
