# Supplementary material for: The Synthesis, Structure, and Properties of a Polynitro Energetic Complex with a Hexaamminecobalt(III) Ion as a Stabilizing Core
Source: Materials (Basel). 2025 Jun 25;18(13):3004. doi: 10.3390/ma18133004 (PMC12250805; doi:10.3390/ma18133004)
Supplement: Supplementary file 1 [file materials-18-03004-s001.zip › materials-3681554-supplementary.pdf]

Supporting information

Synthesis, Structure, and Properties of a Polynitro Energetic Complex with Hexaamminecobalt(III) Ion as a Stabilizing Core

Zhiwei He [a*], *Feng Yang* [a*], *Xianfeng Wang* [b], *and Ming Lu* [b]

[a] School of Chemical and Blasting Engineering, Anhui University of Science and Technology, Huainan 232001, China.

[b] School of Chemistry and Chemical Engineering, Nanjing University of Science and Technology, Nanjing 210094, China.

*Corresponding author. E-mail address: yangf9572@163.com (Feng Yang)

751601138@qq.com (Zhiwei He)

Content

1. Experimental sections……………………………………………1
2. Crystallographic data ……………………………………………3
3. IR and NMR spectrum…………………………….................5
4. Calculation method and results ………………………………6
5. Reference ……………………………………………………….8

1 Experimental Sections

Cautions! Strong acids, bases and other hazardous chemicals are used in the chemical reaction process involved in this article. Wearing protective equipment during the entire experiment is strongly recommended.

General methods: Single-crystal X-ray diffraction measurements were conducted on a Bruker Smart Apex II diffractometer using Mo-Kα radiation (λ = 0.71073 Å) with a graphite monochromator at 296 K. NMR spectra were recorded on Bruker AVANCE III 500MHz at 25 ºC. The onset decomposition temperature (DSC) was measured using NETZSCH DSC204 F1 Phoenix differential scanning calorimeter at a heating rate of 5 °C min^{-1} under a dry nitrogen atmosphere. Infrared spectra (IR) were obtained on a ThermoFisher NICOLETIS10 instrument at 25 °C. Elemental analyses of C/H/N were investigated on a ThermoFisher FLASHSMART Elemental Analyzer (by multiple (5 times, 2~50 mg / times) measurements). Impact and friction sensitivities were tested by a BAM fallhammer and friction tester. Densities were determined at room temperature by employing a Micromeritics AccuPyc 1340 gas pycnometer.

1.1 Synthesis of 5-nitro-3-(trinitromethyl)-1,2,4-triazole (HNTF)

At 25 °C, 50 mL of deionized water was added to a 100 mL three-necked flask. Then 10 g of sodium nitrite (144.9 mmol) was dissolved in it. The mixture was heated to 40 ℃. 4.26 g of 2-(5-amino-1H-1,2,4-triazol-3-yl)acetic acid (30 mmol) was dissolved in 25 mL of dilute nitric acid (8 mL of 68% nitric acid and 17 mL of deionized water). The prepared nitric acid solution was transferred to a constant-pressure dropping funnel and then slowly added dropwise to the sodium nitrite solution. After the addition, the temperature was slowly raised to 50 °C and the reaction was carried out for 2 h. After cooling to room temperature, 0.1 g of urea was added to the reaction system and stirred for 30 min. The mixture was extracted with ethyl acetate. The organic phase was washed with saturated brine, dried over anhydrous magnesium sulfate, and finally distilled under reduced pressure to obtain 3.02 g of a yellow solid (2-(5-Nitro-1H-1,2,4-triazol-3-yl)acetic acid).

In a 50 mL two-necked flask, 12 mL of concentrated sulfuric acid was added, and the temperature was lowered to 0 °C. Then, 10 mL of fuming nitric acid was slowly added dropwise to the concentrated sulfuric acid. Subsequently, 1.29 g of Compound 2-(5-Nitro-1H-1,2,4-triazol-3-yl)acetic acid (15 mmol) was added portionwise to the nitrating mixture of sulfuric and nitric acids. The mixture was slowly warmed to room temperature and reacted at room temperature for 15 h. After completion of the reaction, the solution was poured into 15 g of crushed ice to quench the reaction. After stirring for 2 h, the mixture was extracted with dichloromethane. The organic phase was washed with saturated brine, dried over anhydrous magnesium sulfate, and finally distilled under reduced pressure to yield a white solid.

Yield 0.83 g (46 %). ^{13}C NMR (125 MHz, DMSO-d_6): 157.91, 147.24, 122.27; ^{1}H NMR (500 MHz, DMSO-d_6): 7.20 (s, 1H); IR (KBr): 3113.25, 2947.38, 1632.03, 1601.47, 1570.20, 1524.93, 1479.94, 1443.61, 1369.26, 1278.32, 1174.71, 1080.04, 1044.61, 961.81, 844.89, 827.96, 797.64, 670.72, 645.29, 633.42 cm^{-1}; Element analysis ($C_3N_7O_8H$), calculated (%): C 13.69, H 0.38, N 37.26; measured (%): C 12.96, H 0.91, N 36.68.

1.2 Synthesis of bis(5-nitro-3-(dinitromethyl)-1,2,4-triazole)-hexaamminecobalt(III) [[Co(NH₃)₆](HNTD)(NTD)]

Dissolve 0.526 g (2 mmol) of HNTF in 20 mL of deionized water. Then, add dropwise an aqueous solution of $AgNO_3$ (0.34 g, 2 mmol) in 10 mL of water to the above solution. Stir the mixture in the dark for 12 hours. Filter the mixture and dry the filter cake to obtain the silver salt of HNTF. Thereafter, take 1.11 g (3 mmol) of NTF-Ag and place it in 20 mL of deionized water. While stirring, add dropwise 10 mL of an aqueous solution containing $Co(NH_3)_6Cl_3$ (0.267 g, 1 mmol) to the above suspension. Stir the mixture for 12 hours, then filter it. Slowly evaporate the filtrate to obtain crystalline product $(NTDN)_2[Co(NH_3)_6]$.

Yield 0.652 g (71 %). IR (KBr): 3605.79, 3181.98, 1615.07, 1580.60, 1541.77, 1464.14, 1381.45, 1341.72, 1308.65, 1232.12, 1106.58, 1039.75, 993.51, 839.67, 800.20, 752.08, 724.99, 682.80, 661.96 cm^{-1}.

2 Crystallographic data

Table S1 Crystallography data sheet for compounds **NTNF** and **$(NTDN)_2[Co(NH_3)_6]$**.

**Comp.**	**NTNF**	**$(NTDN)_2[Co(NH_3)_6]$**
Formula	$C_3HN_7O_8$	$C_6H_{21}CoN_{18}O_{13}$
Temperature	296(2) K	293(2) K
System<br>Space group	Monoclinic<br>$P2_1/c$	Monoclinic<br>$P2_1/n$
Dimensions	a = 10.5746(10) Å<br>b = 8.9568(7) Å<br>c = 9.9964(9) Å<br>α= 90°<br>β= 108.817(3) °<br>γ = 90°	a = 10.9674(2) Å<br>b = 13.0975(2) Å<br>c =15.4820(3) Å<br>α= 90 °<br>β= 104.094(2)°<br>γ = 90°
Volume/ $Å^3$	896.20(14)	2156.97(7)
Z	4	4
Density / $g\cdot cm^{-3}$	1.950	1.886
F(000)	528	1256
Theta range / °	3.052 to 27.520	8.942 to 133.168
Index ranges	-13≤h≤12, -10≤k≤11, -12≤l≤12	-13≤h≤11, -15≤k≤15, -16≤l≤18
Reflections collected	9642	28321
Independent reflections	2050<br>[R(int) = 0.0332]	3807<br>[R(int) = 0.0520]
Data/restraints/ parameters	2050 / 0 / 163	3807 / 2 / 351
Goodness-of-fit on F^2	1.040	1.036
Final R indices [I>2sigma(I)]	R1 = 0.0405,<br>wR2 = 0.0924	R1 = 0.0390,<br>wR2 = 0.0964
R indices (all data)	R1 = 0.0622,<br>wR2 = 0.1018	R1 = 0.0423,<br>wR2 = 0.0982
CCDC Num.	2217974	2446547

Table S2. Hydrogen bonds for **NTNF**.

D-H...A	d(D-H)	d(H...A)	d(D...A)	<(DHA)
N(2)-H(2)...N(1)	0.86	2.27	3.086(19)	159.4

Symmetry transformations used to generate equivalent atoms:

#1 x,-y+1/2,z+1/2

Table S3. Hydrogen bonds for **$(NTDN)_2[Co(NH_3)_6]$**.

D-H...A	d(D-H)	d(H...A)	d(D...A)	<(DHA)
N9- H9 ... N3	0.86	1.85	2.678(3)	162.0
N13 - H13A ...O6	0.89	2.38	2.888(3)	116.0
N13- H13A ...O9	0.89	2.33	2.530	144.0
N13 - H13B ...N2	0.89	2.15	3.032(3)	172.0
N13 - H13C ...N8	0.89	2.46	3.188(3)	139.0

O13 - H13D ...O10	0.85	2.27	2.812(4)	121.0
O13 - H13E ...N4	0.85	2.11	2.903(3)	156.0
N14 - H14A ...O5	0.89	2.08	2.912(3)	155.0
N14 - H14B ...N2	0.89	2.39	3.270(4)	168.0
N14 - H14C...O13	0.89	2.16	3.016(3)	160.0
N14 - H14C...O10	0.89	2.42	2.944(4)	118.0
N15 - H15A ...O8	0.89	2.47	3.310(4)	158.0
N15 - H15B ...O13	0.89	2.32	2.974(3)	130.0
N15 - H15C ...O3	0.89	2.11	2.976(3)	163.0
N16 - H16A ...O6	0.89	2.25	3.058(3)	150.0
N16 - H16B ...O3	0.89	2.19	3.045(4)	161.0
N17 - H17B ...O9	0.89	2.26	3.112(3)	160.0
N17 -H17C...O11	0.89	2.54	3.106(3)	122.0
N17 - H17C ... N10	0.89	2.45	3.309(3)	162.0
N18 - H18A ...O4	0.89	2.36	3.022(3)	131.0
N18 - H18B...O4	0.89	2.49	3.302(3)	152.0
N18-H18C...O7	0.89	2.38	3.168(3)	148.0
N18-H18C...O12	0.89	2.57	3.171(3)	126.0

Symmetry transformations used to generate equivalent atoms:

1-x,2-y,-z; # -1/2+x,3/2-y,-1/2+z; #-1/2+x,3/2-y,-1/2+z; # 3/2-x,1/2+y,1/2-z; # 1/2-x,1/2+y,1/2-z # -1/2+x,3/2-y,1/2+z; # -1+x,y,z; # 1/2-x,-1/2+y,1/2-z; # 1-x,1-y,-z; # 3/2-x,-1/2+y,1/2-z; #1/2+x,3/2-y,1/2+z; # 1-x,1-y,-z;# 1/2+x,3/2-y,1/2+z; # 1/2+x,3/2-y,1/2+z; # 1+x,y,z; #3/2-x,1/2+y,1/2-z; # 1/2+x,3/2-y,-1/2+z; # 1-x,1-y,-z; # 3/2-x,-1/2+y,1/2-z

IR and NMR spectrum

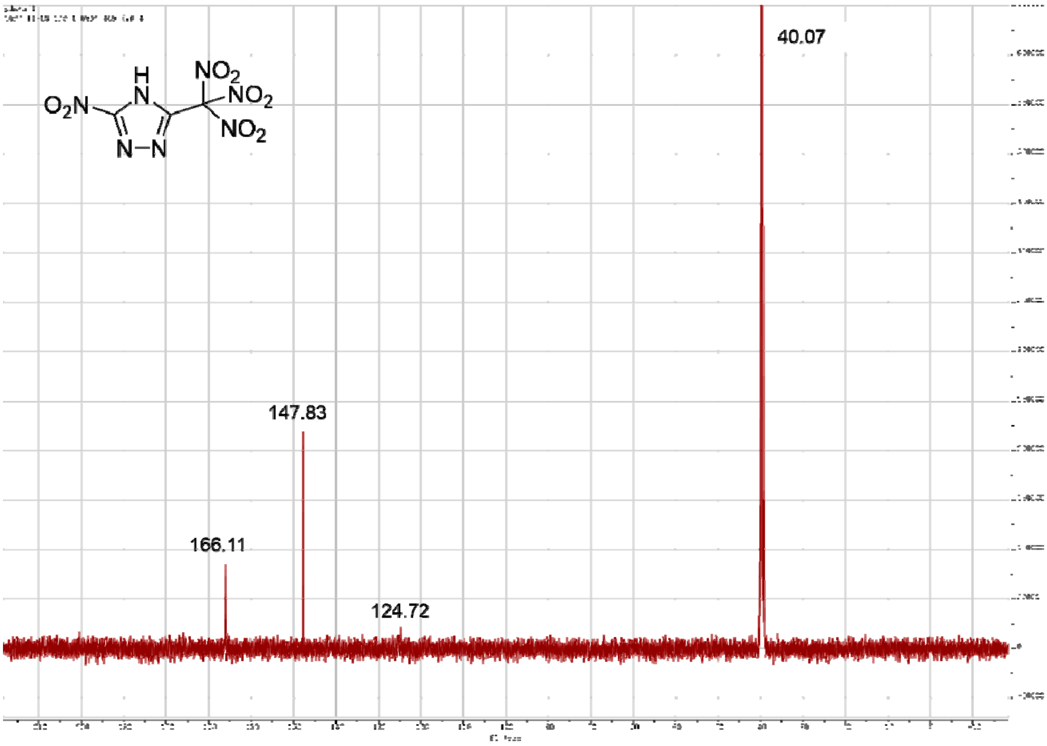

Figure S1. ^{13}C NMR of NTNF (^{13}C NMR, DMSO-d_6).

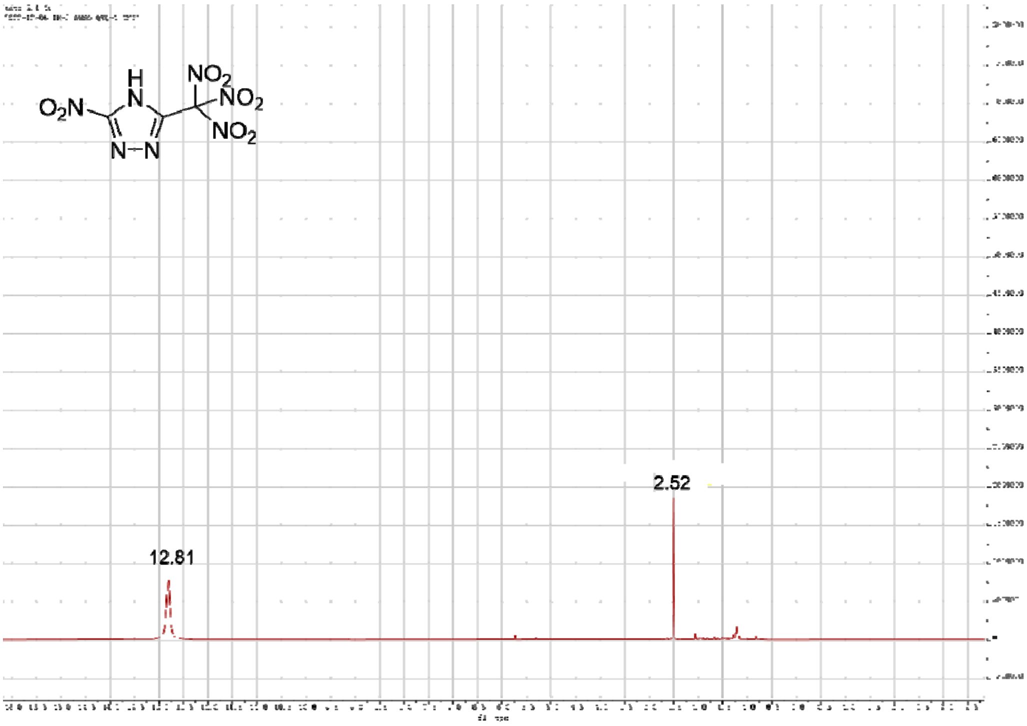

Figure S2. ^{1}H NMR of NTNF (^{1}H NMR, DMSO-d_6).

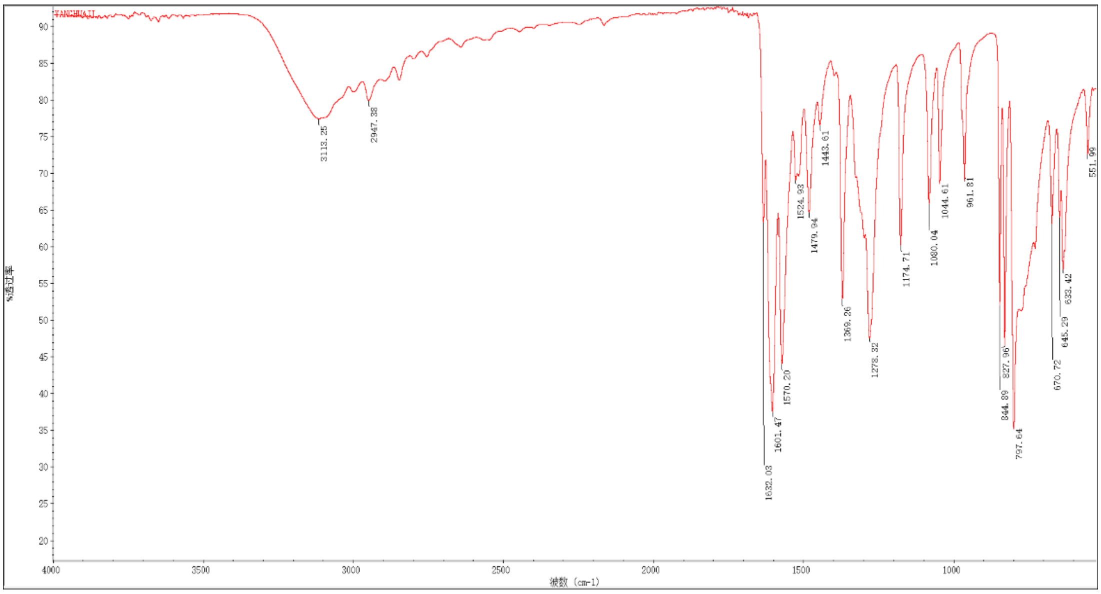

Figure S3. IR of NTNF.

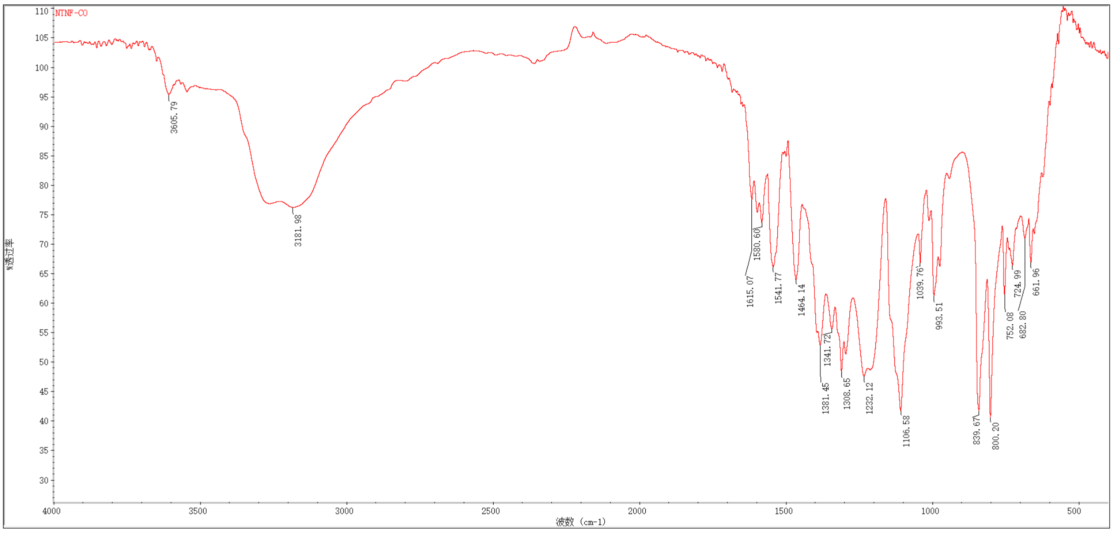

Figure S4. IR of $(NTDN)_2[Co(NH_3)_6]$.

Calculation method and results

The geometric optimization and frequency analyses of all these compounds were based on available single-crystal structures by Gaussian 09 suite[1] of programs and using the B3LYP functional with the 6-311G(d,p) basis set. The geometrical configurations were optimized with no constraints imposed under default convergence criteria. All of the optimized structures were characterized to be true local energy minima on the potential energy surface without imaginary frequencies. Thermal corrections to enthalpy were computed at the same DFT level of theory. The gas phase heats of formation were calculated using isodesmic reactions (Figure S5). [2]

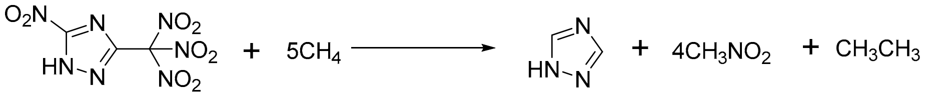

Figure S5 Isodesmic-reaction of **NTNF**.

The enthalpy of sublimation can be represented as eq (1)[3] and on the basis of thepredicted electrostatic potential of a molecule.[4]

$$\Delta H_{sub} = a(SA)^2 + b\sqrt{\upsilon\sigma_{tot}^2} + c \qquad (1)$$

Here SA is the surface area of the 0.001 electrons bohr^{-3} isosurface of the electronic density of the compounds, $\upsilon\sigma_{tot}^2$ is derived from the molecular electrostatic potential calculation, and a, b, c are fitting parameters reported by Politzer et al.[4]

Table S4. Calculated zero-point energy (ZPE), thermal correction to enthalpy (ΔH_T), total energy (E_0) and gas phase heats of formation (HOF)

Compound	*ZPE* / kJ mol^{-1}	ΔH_T / a. u.	E_0 / a. u.	*HOF* / kJ mol^{-1}
**NTNF**	251.55	0.111932	-1099.7843702	123.2
CH_4	118.22	0.048836	-40.5240195	-74.60
Triazole	157.45	0.064481	-242.249273	237.91
CH_3NO_2	131.32	0.055294	-245.013375	-74.30
CH_3CH_3	73.092	0.004484	-79.8416813	-84.21

The constant pressure reaction heat ($\Delta_c U$) of $(NTDN)_2[Co(NH_3)_6]$was measured by an oxygen bomb calorimeter, and the average value was obtained by three measurements independently. The standard molar combustion enthalpy ($\Delta_c H_m^\theta$) can be obtained from the constant pressure reaction heat ($\Delta_c U$) according to the equation 1 and the complete combustion reaction equation of $(NTDN)_2[Co(NH_3)_6]$ is as shown in equation 2 According to the principle of Hess' law, the standard molar generation enthalpy ($\Delta_c H_m^\theta$) can be obtained based on the formulas 2 and 3 (CO_2(g): -393.51 kJ mol^{-1}; CoO(s): - 237.74 kJ mol^{-1}; H_2O(l): -285.85 kJ mol^{-1}).

$$\Delta c H_m^\theta = \Delta c U + \Delta n RT \qquad \text{(S1)}$$

Δn = n_g(products)-n_g(reactants), (n_g is the sum of the total moles of gas in the product or reactant, R = 8.314 J mol^{-1} K^{-1}, T = 298.15 K)

$$C_6H_{21}CoN_{18}O_{13} + 5O_2 = 6CO_2 + 10H_2O + CoO + 9N_2 \qquad \text{(S2)}$$

$$\Delta_f H_m^\theta(compound) = \sum \Delta_f H_m^\theta(products) - \Delta_c H_m^\theta(compound) \qquad \text{(S3)}$$

Detonation velocity (D) and explosion pressure (P) are the main indicators for measuring energetic materials. The various detonation characteristics of the ECPs were predicted using the modified Kamlet-Jacbos (K-J) equations (eq 4-6) which is a commonly used equation for predicting the detonation velocity and pressure of high energy materials.

$C_6H_{21}CoN_{18}O_{13}$ =CoO + 10.5H_2O + 1.5CO + 4.5C + 9N_2

$$D = 1.01(NM^{1/2}Q^{1/2})^{1/2}(1+1.30\rho) \quad \text{(S4)}$$

$$P = 1.55\rho^2 NM^{1/2}Q^{1/2} \quad \text{(S5)}$$

formula weight of explosive

$$Q = \frac{-[\Delta_f H(detonationproduction) - \Delta_f H(\exp losive)]}{formulaweight} \quad \text{(S6)}$$

D: detonation velocity, km s^{-1}); P: detonation pressure, GPa; ρ: density, g cm^{-3}; $\Delta_f H$: heat of formation, kJ mol^{-1}); Q: heat of detonation, J g^{-1}); N: moles of detonation gases per gram of explosive, mol g^{-1}); M: average molecular weight of gases, g mol^{-1}).

Reference

(1) M. J. Frisch, Gaussian 09. Revision a. 02, Gaussian, Inc., Wallingford CT, 2009.

(2) J. Zhang, H. D, F. Wang, DFT Studies on a High Energy Density Cage Compound 4-Trinitroethyl-2,6,8,10,12-pentanitrohezaazaisowurtzitane, *J. Phys. Chem. A*, 2011, **24**, 6617-6621.

(3) P. W. Atkins, Physical Chemistry. Oxford University Press, Oxford, U. K., 1982.

(4) P. J. Politzer, S. Murray, T. Brinck, P. Lan, Immunoanalysis of agrochemicals. ACS Sympsium Series 586, American Chemical Society, Washington, DC, 1994.
